# Rapid Analysis of α-Tocopherol and Its Oxidation Products Using Supercritical Carbon Dioxide and Proton Transfer Reaction Ionization Mass Spectrometry

**DOI:** 10.5702/massspectrometry.A0108

**Published:** 2022-12-20

**Authors:** Chihiro Ota, Toshinobu Hondo, Yumi Miyake, Hiroshi Furutani, Michisato Toyoda

**Affiliations:** 1 Graduate School of Science and Engineering, Kansai University, 3–3–35 Yamate-cho, Suita, Osaka 564–8680, Japan; 2 Forefront Research Center, Graduate School of Science, Osaka University, 1–1 Machikaneyama, Toyonaka, Osaka 560–0043, Japan; 3 MS-Cheminformatics LLC, Sasao-nishi 2–13–21, Toin, Inabe, Mie 511–0231, Japan; 4 Center for Scientific Instrument Renovation and Manufacturing Support, Osaka University, 1–2 Machikaneyama, Toyonaka, Osaka 560–0043, Japan

**Keywords:** supercritical fluid extraction-supercritical fluid chromatography, α-tocopherol, antioxidant, proton-transfer reaction, chemical ionization

## Abstract

We have developed a rapid and sensitive analytical method for α-tocopherol and its oxidative products by combining online hyphenation of supercritical fluid extraction-supercritical fluid chromatography (SFC) with proton transfer reaction (PTR) ionization mass spectrometry (MS). α-Tocopherol is a well-known antioxidant that plays a vital role in the antioxidant defense system in plant cells. However, studies on the cellular mechanisms of α-tocopherol have been limited owing to the lack of a rapid analytical method, which limits the comparison of plant cells incubated in various conditions. Additionally, complex sample preparation and long chromatography separation times are required. Moreover, the majority of the involved molecules are a combination of isomers, which must be separated before applying tandem MS. α-Tocopherol produces the α-tocopheroxyl radical in the first step of its antioxidant function; another ion with the same mass may also be generated from the source. SFC separation effectively distinguished the observed ions from their oxidative products in the sample and those produced during the ionization reaction process. This method enabled the measurement of α-tocopherol and its oxidative products such as α-tocopheroxyl radical and α-tocopheryl quinone in approximately 3 min per sample, including the time required for sample preparation.

## 1. INTRODUCTION

Since its development by Lindinger *et al.*^1)^ in the late 1990s, proton transfer reaction mass spectrometry (PTR-MS) has been used as an effective method for measuring volatile organic compounds (VOCs) in gaseous samples. It has been used in various applications involving the measurement of VOCs in volcanic gases and forest fires, metabolite analysis of breath, identification of microorganisms, *etc*.^2–6)^ Recently, we have invented the simplified ion source inspired by PTR-MS, which uses a mixture of water-positive and negative ions as reagent ions. Those ions react with analytes so that the proton transfer reaction occurs in protonated and deprotonated directions, which is slightly different from the traditional PTR-MS. Although it is essentially a water ion chemical ionization, we have called it proton transfer reaction (PTR) ionization mass spectrometry (MS).^7,8)^

Supercritical fluid extraction (SFE) is a rapid and safe method for extracting lipophilic compounds from complex sample matrices. When applicable, SFE can also be used to extract polar molecules employing an entrainer. SFE was developed by Zosel in the 1970s^9)^ and has been applied in the industrial-scale extraction of food and fine chemical industries because carbon dioxide, the most common fluid for SFE, is safe to use in foods. A supercritical fluid selectively extracts compounds depending on the temperature, pressure, and entrainer (such as ethanol) that can transport various molecules to another vessel, making it suitable for industrial-scale extraction and analytical-scale sample preparation.

Because supercritical fluids have a lower viscosity than liquids and an almost identical density to liquids, supercritical fluid chromatography (SFC) exhibits a higher separation efficiency than liquid chromatography (LC). Thus, SFC has also been used for the optical peak resolution (*R*s) of racemic compounds^10)^ and steroids.^11)^ In addition to separation efficiency, the selectivity (α) of SFC can change the pressure and temperature of the mobile phase, which gives more flexibility to optimize the peak resolution of racemic compounds than LC. Unique applications such as bioactive lipids in aqueous solution have been reported^12)^ by combining SFE and SFC. Recently, noteworthy advances in fluid control technology have afforded new developments such as ultrafast separation using SFC.^13,14)^

**Figure 1:**
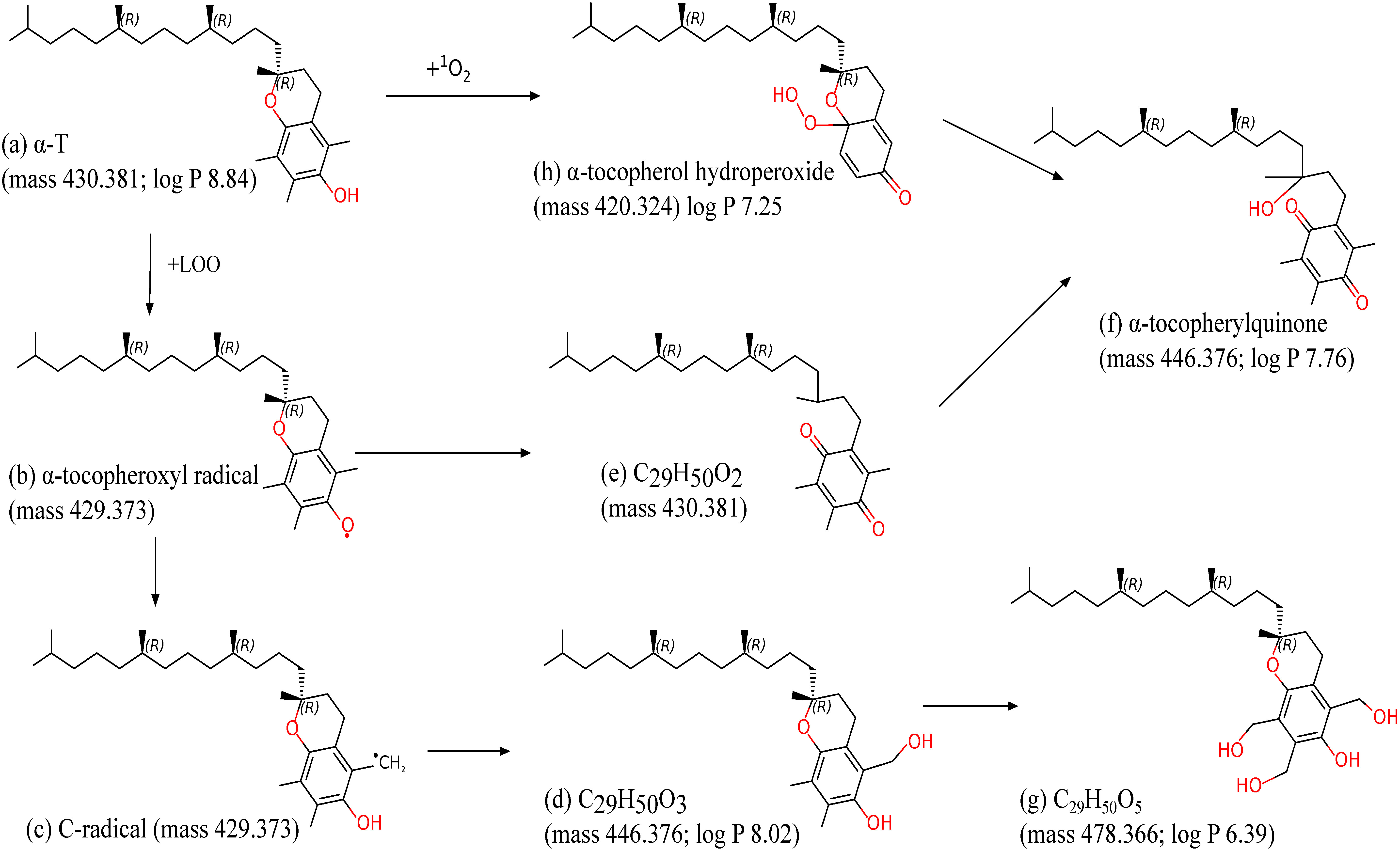
Fig. 1. Oxidative reaction schematic of α-tocopherol described in the literature. The log *P* values were calculated using a function on the rdkit.Chem. Crippen module.

Plant cells have several enzymatic and nonenzymatic self-defense mechanisms against oxidation. α-Tocopherol (α-T), in particular, is well known to prevent lipid peroxidation.^15–17)^ The α-T oxidation reaction scheme reported by Kumar *et al.*^18)^ and Tang *et al.*^19)^ is summarized in Fig. 1. The log *P* values shown in the figure were calculated using a function on the rdkit.Chem Crippen module.^20)^ Kumar *et al.* investigated the formation of α-tocopheroxyl radical and α-T-hydroperoxide by thylakoid membrane and rose bengal as the source of singlet oxygen (^1^O_2_). They used a combination of analytical instruments, including LC with a photodiode array (PDA) detector, fluorescence spectroscopy, and electron paramagnetic resonance spectroscopy to detect α-T hydroperoxide, α-T radicals, and ^1^O_2_. A crucial function of α-T is quenching ^1^O_2_ before the oxidation of fatty acids. Tang *et al.* reported the detailed chemical structures of α-T oxidization products by monitoring changes in ultraviolet absorption spectra using LC–PDA detection and analyzing the corresponding chromatographic peaks *via* quadrupole time-of-flight (TOF) mass spectrometer.

Although the details of the self-defense system of plant cells against ^1^O_2_ using α-T are recently being determined by combining several analytical methods, the analytical process involves complex procedures and requires more than 30 min of chromatographic run time. Accordingly, a more rapid and sensitive method for investigating the defense system in individual cells is required. Additionally, because ambient autooxidation occurs during sample preparation, sample processing must be completed as quickly as possible to avoid possible artifacts.

As previously reported,^7,8)^ SFE–PTR MS can also detect nonvolatile small molecules with high sensitivity, at least up to *m*/*z* 1000. The addition of chromatography separation to SFE–PTR MS improves the analytical performance, such as isomer peak resolution and better detection sensitivity. We further evaluation of our PTR flow tube prototype revealed approximately two seconds of longitudinal dispersion, which is practically applicable to the SFC and affords an overall narrow peak width. In this study, we have investigated a preparation-free rapid analysis of α-T and its oxidative products using SFE/SFC–PTR MS.

## 2. EXPERIMENTAL

### 2.1. Chemicals

α-T, hexamethoxyphosphazine (P321), hexakis (2,2-difluoroethoxy) phosphazene (P621), ethanol, and acetonitrile (LC/MS grade) were purchased from FUJIFILM Wako Pure Chemicals Corporation, Osaka, Japan. Water was obtained from a Milli-Q purification system (Merck, Germany). Cylinders of general-grade helium and carbon dioxide (siphon type) (Iwatani Industrial Gases Corp, Osaka, Japan) were used.

The purchased α-T was stored in the dark at −80°C until use. A 10-μM α-T solution was prepared in 1 mL acetonitrile and used without further purification after incubation at room temperature (25°C) in Eppendorf sample tubes for a given period.

InertSustain C18 (3 μm; 1.0-mm inner-diameter (i.d.)×30-mm length; GL Science, Tokyo, Japan; InertSustain) and L-column3 C18 (3 μm; 2.1-mm i.d.×100-mm length; CERi, Chemicals Evaluation and Research Institute, Tokyo, Japan; L-column3) were used for SFC separation.

### 2.2. Mass spectrometer

With a minor modification, a JMS-T100 LP (AccuTOF) TOF mass spectrometer (JEOL, Tokyo, Japan) was used with an Acqiris Model U5303A (3.2 GS·s^−1^ 12-bit digitizer, Geneva, Switzerland) as the data acquisition system. Data acquisition was performed using an open-source software “QtPlatz” (https://github.com/qtplatz/qtplatz) with a modified field programmable gate array configuration for acquiring peak detection (PKD) and waveform averaging (AVG) simultaneously.^21)^ The PKD histogram and AVG waveform were collected every 200 ms using U5303A.

**Figure 2:**
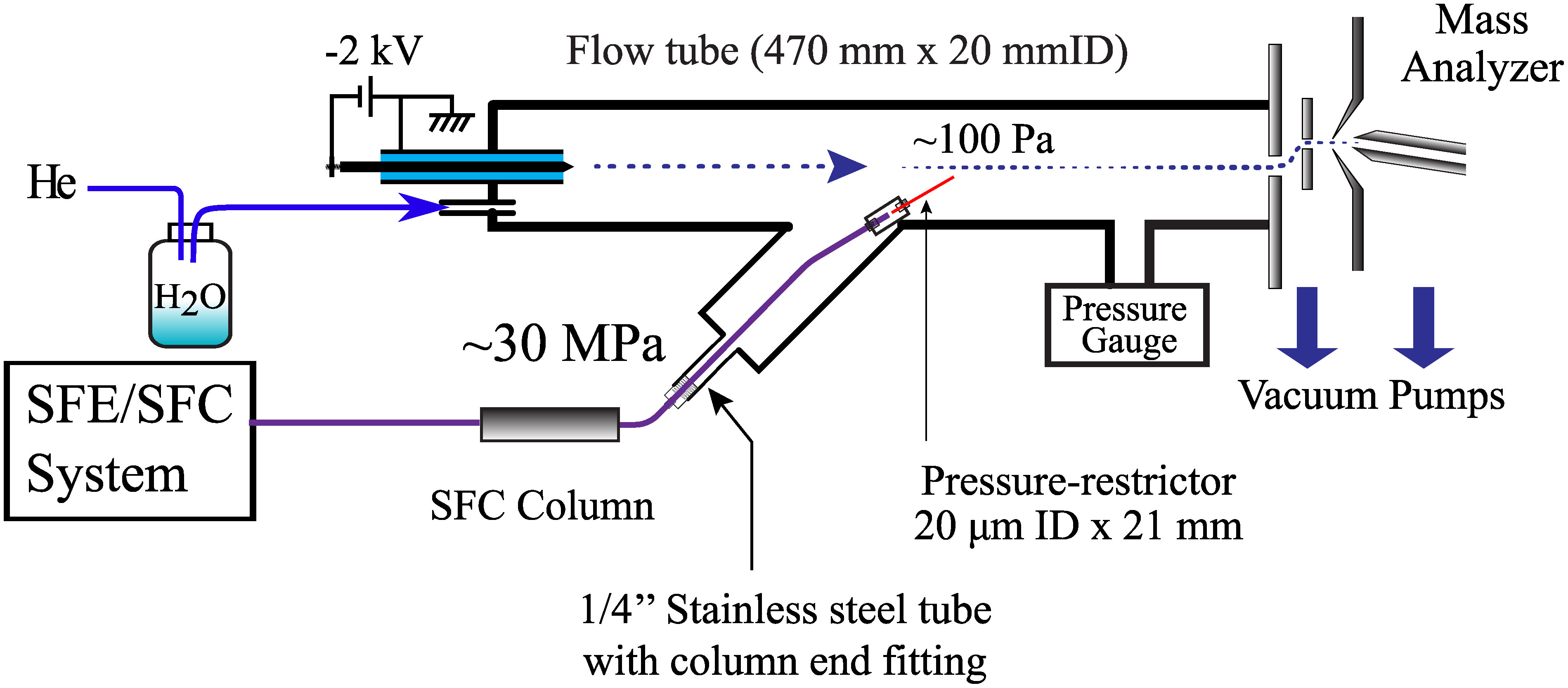
Fig. 2. Schematic of the PTR flow tube.

Figure 2 shows the block diagram of the PTR ion source, comprising a flow tube with a corona discharge electrode. Before entering the flow tube, helium was allowed to flow through a 250-mL solvent-reservoir bottle, containing about 10 mL of ultrapure water (Millipore, MA, US). The helium flow rate was set to 70 mL·min^−1^, which was controlled *via* a mass flow controller (8500MC-0-1-2, KOFLOC Corp., Kyoto, Japan). An AccuTOF vacuum system maintained pressure in a range of 90 to 105 Pa in the flow tube. A PS350 high-voltage power supply (Stanford Research Systems, Inc., Sunnyvale, CA, US) was used as a corona discharge power supply. The voltage for the discharge electrode was set to −2.0 kV, generating a current of 80–100 μA.

### 2.3. SFE/SFC splitless injection

**Figure 3:**
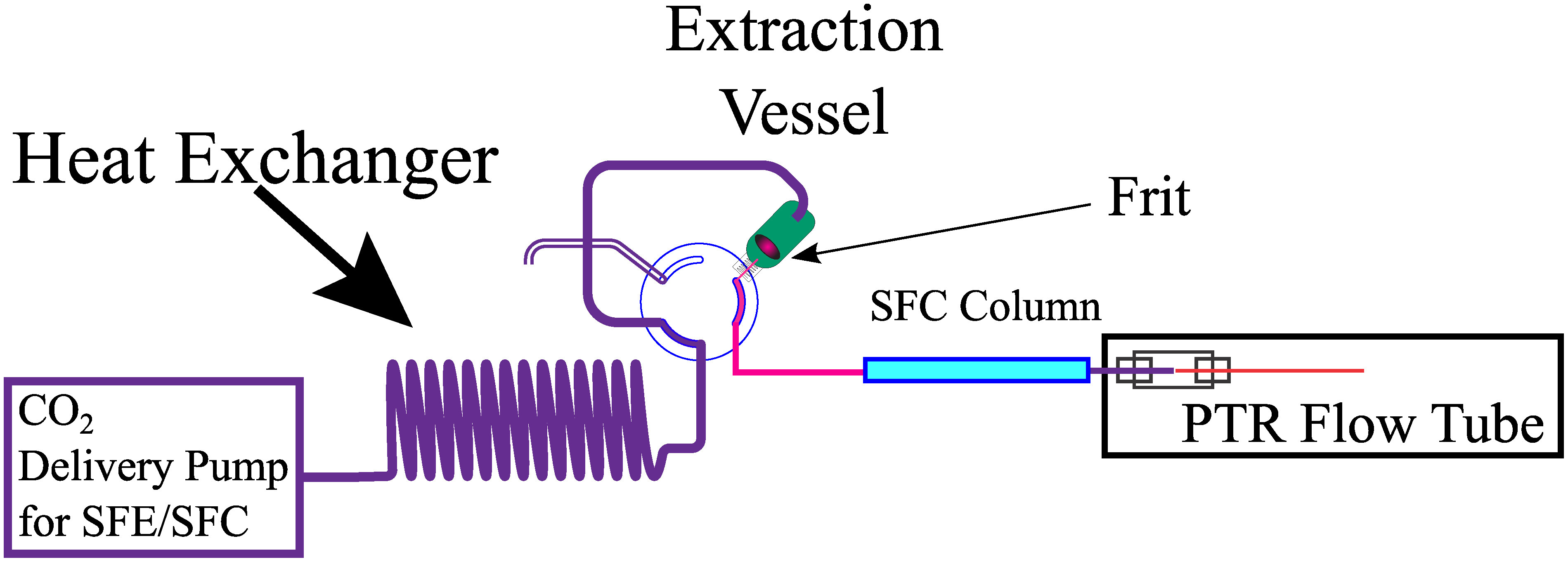
Fig. 3. Hydraulics of SFE/SFC with a splitless injection.

Figure 3 shows the hydraulics of a splitless injection. Liquid carbon dioxide from the cylinder was precooled to −5°C in an ethanol/dry-ice bath and delivered at a rate of 0.5 mL·min^−1^ at 25 MPa using an LCPackings UltiMate Micropump (Thermo Scientific, US), equilibrated at 40°C in an oven (Agilent 1100 Series Thermostatted Column Compartment; Agilent, CA, US) and connected to an SFC column (InertSustain). As a pressure restrictor, the outlet of the column was connected to a PTR flow tube that passed through a 20-μm i.d. × 21-mm length fused silica capillary (GL Science, Tokyo, Japan).

The sample dissolved in acetonitrile was applied to a stainless steel frit, and acetonitrile was allowed to evaporate. The frit with the sample was then attached to an in-line filter (ACQUITY Column In-Line Filter (Waters, US)) and introduced into the SFC flow by switching the valve (Rheodyne 7000, Rheodyne, USA) position.

### 2.4. SFE/SFC split injection

**Figure 4:**
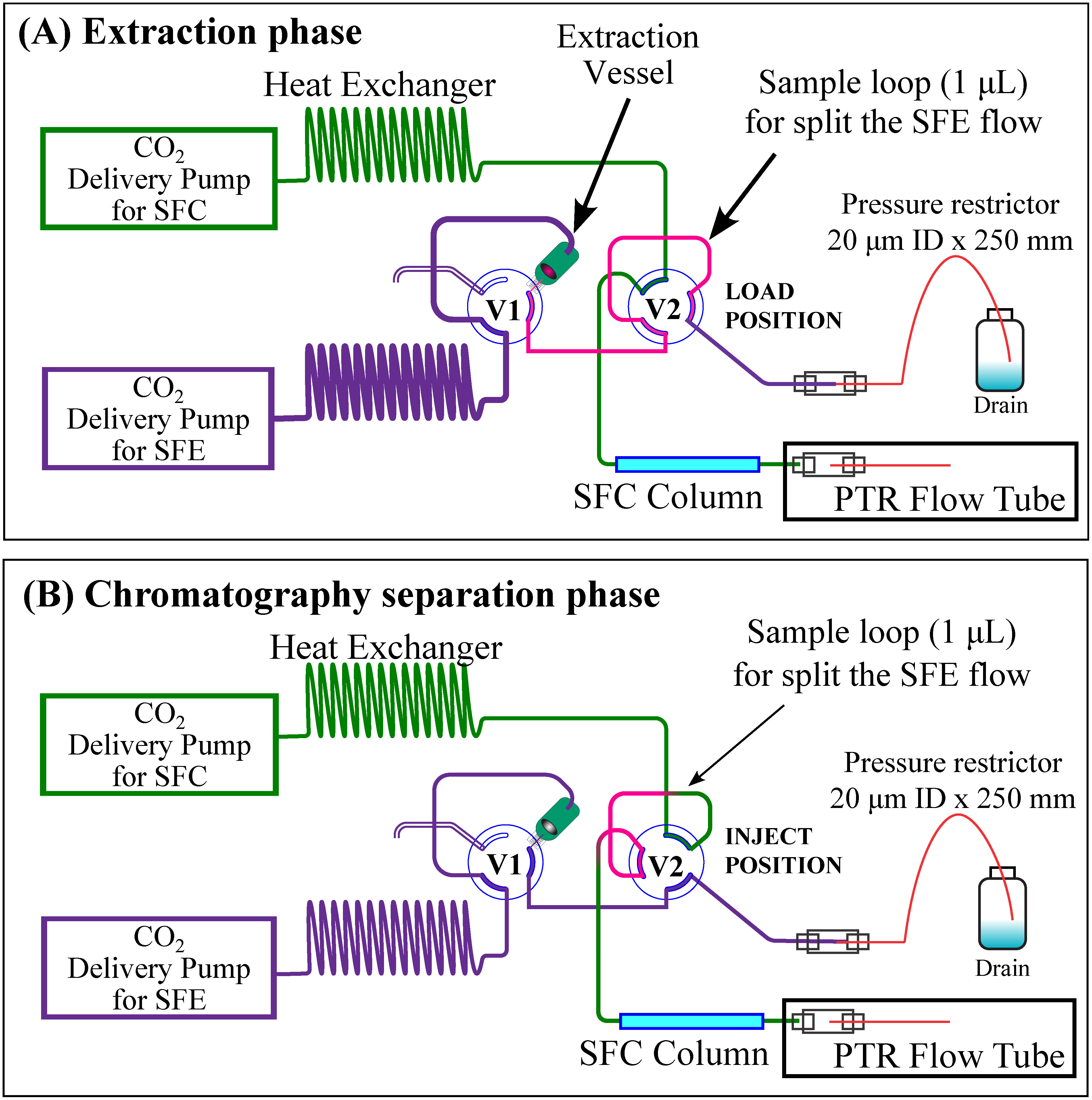
Fig. 4. Hydraulics of SFE/SFC with a split injection.

Figure 4 shows the hydraulics of an SFE/SFC split injection, in which SFE and SFC have separate carbon dioxide delivery systems. In the SFE hydraulics, liquid carbon dioxide was precooled to −5°C in an ethanol/dry-ice bath and delivered at a rate of 200 μL·min^−1^ at a pressure of 25 MPa using an LCPackings UltiMate Micropump. Carbon dioxide from the pump was equilibrated at 40°C in an oven and then passed through Rheodyne 7000 switching valves V1 and V2. Carbon dioxide was discharged using a pressure restrictor (20-μm i.d. × 250-mm length).

In the SFC hydraulics, liquid carbon dioxide was precooled to −5°C in an ethanol/dry-ice bath and delivered at a rate of 1.0 mL·min^−1^ at 30 MPa using a PU-980 pump (JASCO, Tokyo, Japan). Carbon dioxide from the pump was equilibrated at 40°C in an oven, and then the supercritical carbon dioxide was connected to the separation column *via* valve V2. The mobile phase from the column was connected to a pressure restrictor in the PTR flow tube. This method used either an InertSustain or an L-column3 column for SFC separation. SFC hydraulics shared V2 with SFE hydraulics, in which 1 μL of fluid was divided and introduced into the column. The carbon dioxide flow rate, temperature, pressure, and timing of the split flow at the beginning of SFE may affect the composition and amounts of the sample to be injected.

All pump heads for carbon dioxide delivery were cooled to approximately 8°C using a Peltier module (TES112705, Hebei, China).

### 2.5. Mass calibration

Mass calibration was performed using sodium trifluoroacetate, an electrospray ionization (ESI) source for *m*/*z* between 159 and 703, and third-order polynomials. The PTR ion source was then attached by replaced with the ESI source.

## 3. RESULTS AND DISCUSSION

### 3.1. SFE/SFC analysis of α-T incubate

**Figure 5:**
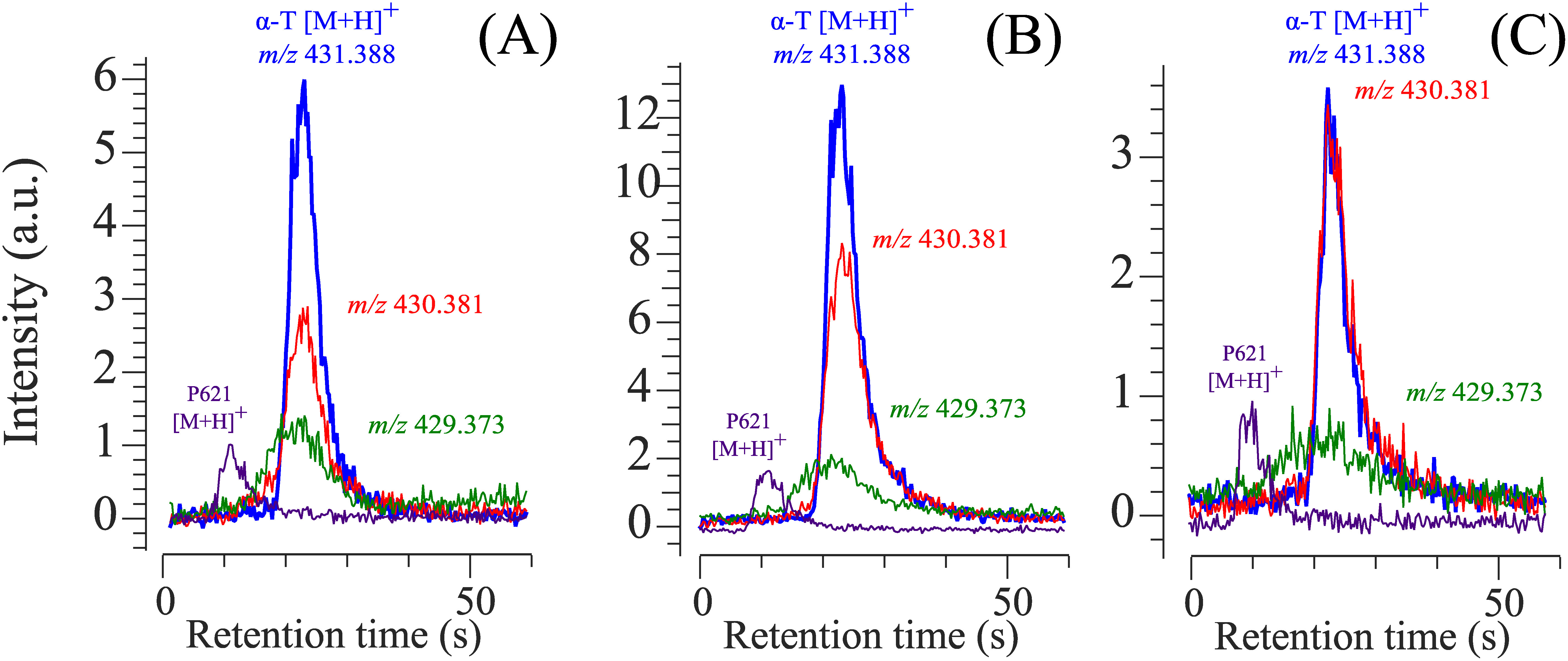
Fig. 5. Extracted ion chromatograms of α-tocopherol (α-T) and the suspected oxidative products analyzed using a splitless injection. A: freshly prepared sample, B: sample incubated for 1 day at the ambient temperature (approximately 25°C), and C: sample incubated for 7 days at the ambient temperature. Each of the 5 pmol of the α-T sample was applied on the frit. SFC condition: InertSustain C18; mobile phase: carbon dioxide, 40°C, 0.5 mL min^−1^, 25 MPa isobaric.

The α-T incubates obtained after 0, 1, and 7 days of incubation at ambient temperature (≈25°C) were analyzed using SFE/SFC splitless injection for all the 5 pmol of α-T. Figure 5 shows the extracted ion chromatograms obtained for *m*/*z* 431.388, 430.381, and 429.373, corresponding to [M+H]^+^, [M]^+^, and [M−H]^+^ of α-T, respectively. Each chromatogram for *m*/*z* 431.388 presented in Figs. 5 (A), (B), and (C) shows a single chromatographic peak with the highest intensity. The chromatographic peaks shown in Fig. 5 are supposed to be a mixture of the α-T and its oxidative products listed in Fig. 1 and maybe molecules uncovered yet. The relative intensity of the chromatographic peak at *m*/*z* 430.381 drastically changes during the incubation period. Although the chromatogram for *m*/*z* 429.373 shows a broad peak on all three samples, the relative retention time to the chromatographic peak for *m*/*z* 431.388 appears to decrease. As shown in Table 1, the relative peak area of chromatograms for *m*/*z* 430.381 compared with 431.388 increased by 1.45 and 2.20 folds for 1 and 7 days of incubation, respectively. Although the relative peak area of chromatograms for *m*/*z* 430.381 changed considerably, no apparent changes in chromatographic properties, such as peak resolution or retention factor, were observed. Figure 1 suggests that in the protonated form, several reported oxidative products could exhibit peaks at *m*/*z* 430.381 and 431.388; the obtained chromatographic peak comprises a couple of coelutes. To resolve these molecules, a better chromatography separation technique is required.

**Table 1:** Table 1. Peaks obtained from the chromatograms presented in Fig. 5.

	*m*/*z*	*t*_R_(s)	Area	Height	Width(s)	*t*_R_/*t*_R_ (431)	Area ratio (A/A_431_)	Fold
A	622.029	11.2±0.6	9.0±2.6	1.45	4.07			
	431.388	23.1±0.5	34.4±2.0	5.38	5.04	1.00	n/a	n/a
	430.381	23.0±0.4	18.8±4.5	2.34	5.74	1.00	0.543	100.0%
	429.373	22.6±1.5	12.03±0.01	1.25	6.58	0.98	0.351	100.0%
B	622.029	11.7±0.6	10.72±0.68	1.73	4.79			
	431.388	22.9±0.2	58.3±26.3	9.24	5.25	1.00	n/a	n/a
	430.381	23.3±0.3	48.4±26.5	6.22	6.63	1.02	0.810	149.2%
	429.373	22.5±0.5	18.7±8.6	1.60	6.71	0.98	0.319	91.0%
C	622.029	11.4±0.3	3.84±0.97	0.98	2.27			
	431.388	25.6±1.8	15.67±3.11	3.11	4.13	1.00	n/a	n/a
	430.381	25.8±1.7	18.81±3.02	3.02	4.94	1.05	1.196	220.2%
	429.373	22.4±3.6	7.92±2.97	0.65	2.61	0.87	0.497	141.8%

The volume of an extraction vessel is approximately one microliter, which is small enough to form a narrow peak bandwidth that is compatible with SFC. However, chromatographic peaks were obtained with a peak width of 2.3–6.8 s (Table 1), which were derived from the SFE profile with additional molecule-specific extraction delay. Each molecule is injected into the column with its own bandwidth by slightly separated SFE profiles, which results in each molecule showing a few seconds of peak width. Split injection is a simple and effective method for introducing a narrow sample bandwidth to SFC to improve peak resolution by limiting sample bandwidth as well as timing synchronization.

### 3.2. Determination of split timing

**Figure 6:**
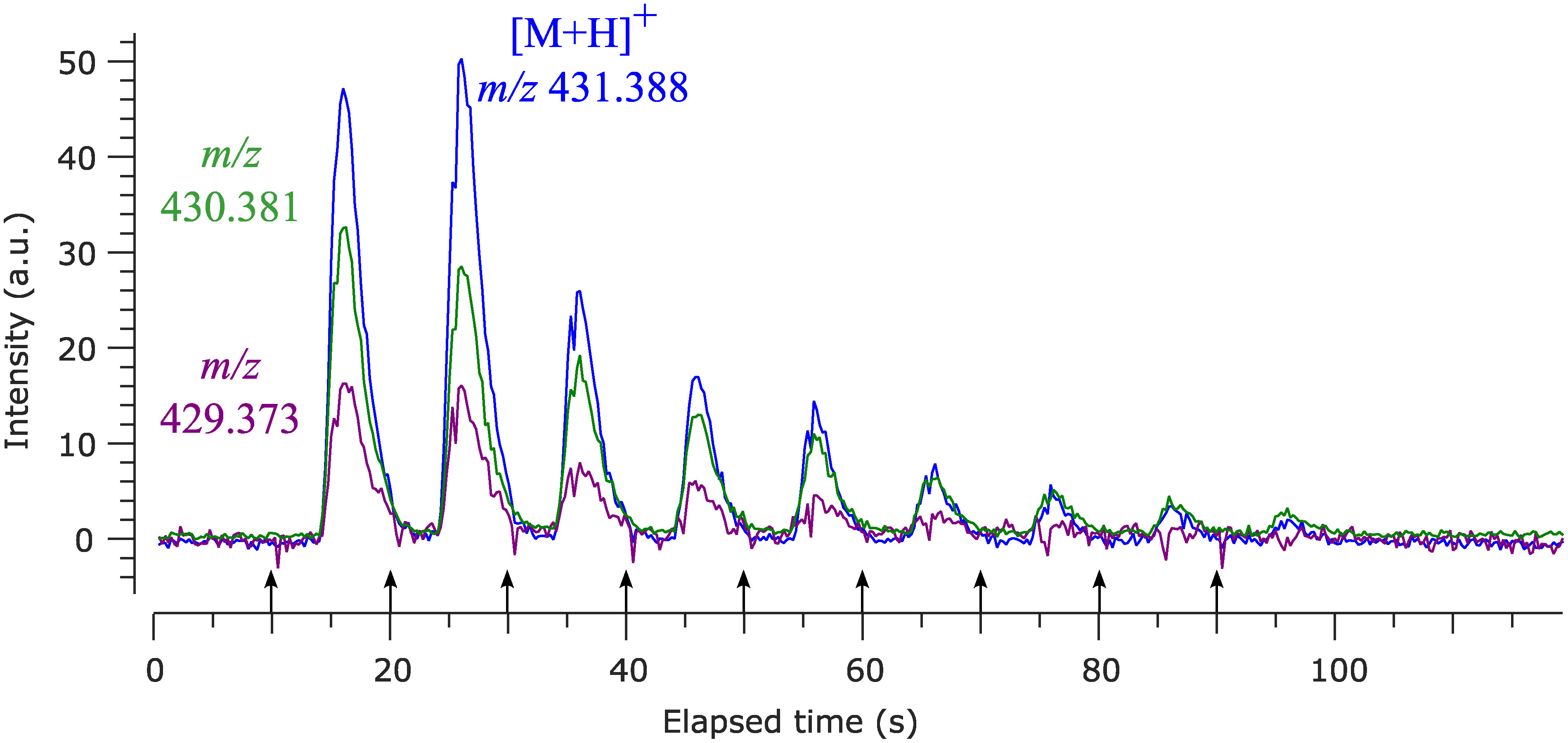
Fig. 6. Determination of split timing to inject a sample in the fluid to SFC after the initiation of SFE. Time course of peak intensity for 1-μL split injection since the beginning of SFE without an SFC column. The upper arrow indicated above the horizontal axis indicates each injection time point.

As described in Section 3.1, SFE extraction profiles show a peak with a width of a few seconds. Finding a peak apex point is optimal for splitting an SFE flow; however, apex timing is dependent on the molecule and SFE conditions. Therefore, experimentally determining optimal timing for the given sample matrix is necessary. Figure 6 shows a series of sample injections splitting SFE flow into SFC hydraulics without using a separation column. An extracted ion profile monitored at *m*/*z* 431.388 provided the best split timing of 20 s, with the most intense peak. However, a profile monitored at *m*/*z* 430.381 and 429.373 produced the most intense peak at 10-s split timing. Because there is no considerable difference between the peak intensity of *m*/*z* 431.388 at 10 and 20 s, we use the 10-s split timing for further experiments.

### 3.3. Comparison of chromatograms of splitless and split injection

**Figure 7:**
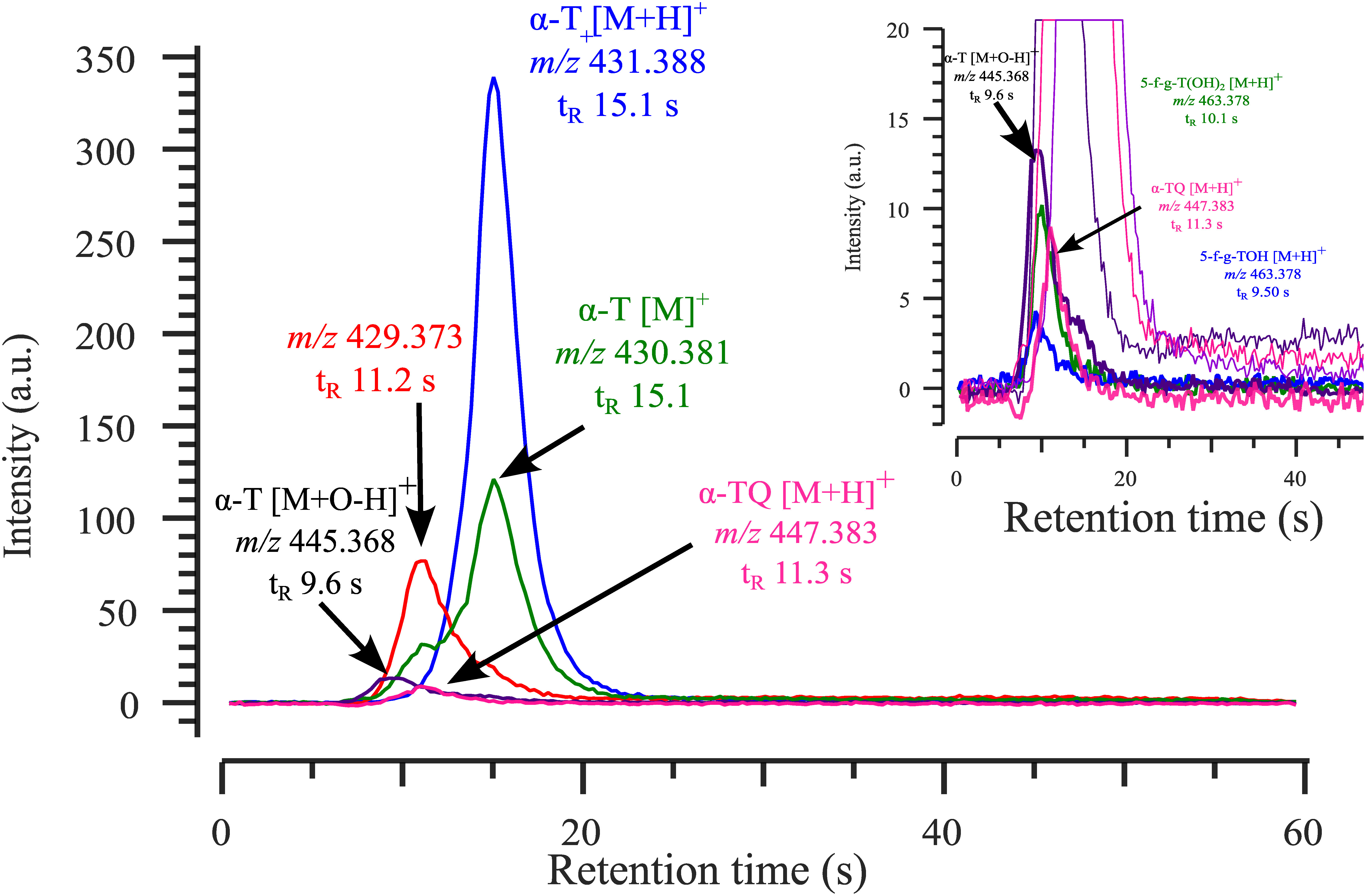
Fig. 7. Extracted ion chromatograms of α-tocopherol (α-T) and suspected oxidative products analyzed using split injection. Ten picomoles of α-T were applied on the frit. The inset is scaled on the vertical axis for visibility. SFE condition: carbon dioxide, 40°C, 25 MPa, 200 μL·min^−1^; SFC condition: InertSustain C18; mobile phase: carbon dioxide, 40°C, 1.0 mL min^−1^, 25 MPa isobaric.

Figure 7 shows a chromatogram of 10 pmol of the α-T sample using split injection, corresponding to Fig. 5 (B), which is a 1-day incubated α-T sample. The extracted ion chromatogram for *m*/*z* 431.388 shows a single peak with a retention time of 15.1 s and a width of 2.7 s, which is half the peak width obtained using splitless injections. A chromatogram for *m*/*z* 430.381 exhibited a peak with the same retention time *m*/*z* 431.388; however, it also showed an unresolved shoulder peak at 11.2 s. Sample amounts applied to the frit for split injection were 10 pmol, which was double the amounts of splitless injection (5 pmol) chromatogram; however, peak intensities obtained with the split injection were approximately 30 times higher than those obtained with splitless injection. It led us to discover the other oxidative product ions of α-T, including those with *m*/*z* values of 445.368, 463.378, and 447.383, as described in Fig. 1 and the literature.^19)^

### 3.4. Separation of α-T oxidative products

**Figure 8:**
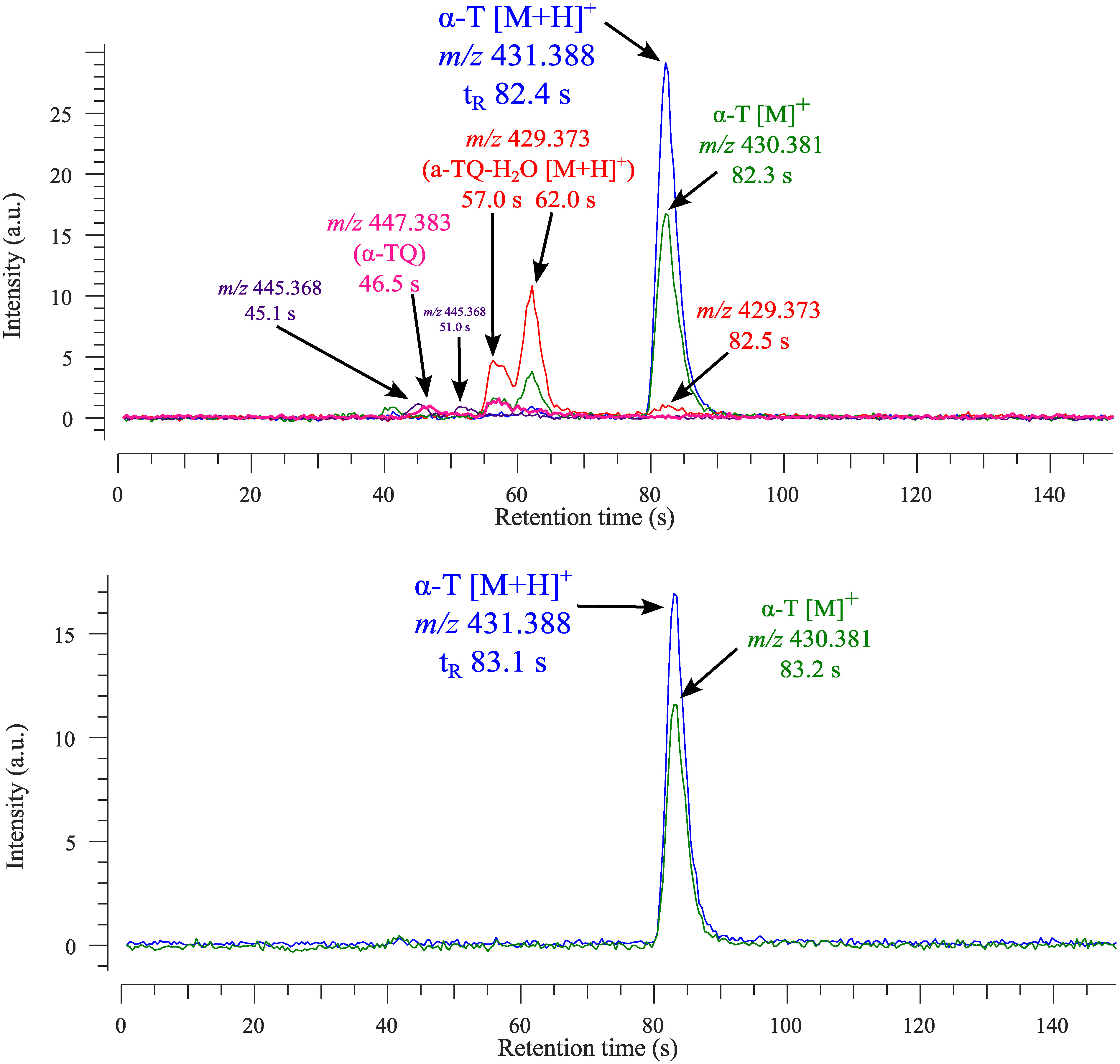
Fig. 8. SFE/SFC split injection chromatograms of α-tocopherol (10 pmol) and suspected oxidative product separated using L-column3 C18 columns. Bottom plots present the freshly prepared samples; top plots present the samples incubated for 21 days at the ambient temperature. SFE condition: carbon dioxide, 40°C, 25 MPa, 200 μL·min^−1^; SFC condition: L-column3 C18; mobile phase: carbon dioxide, 1.0 mL min^−1^, 30 MPa isobaric.

**Figure 9:**
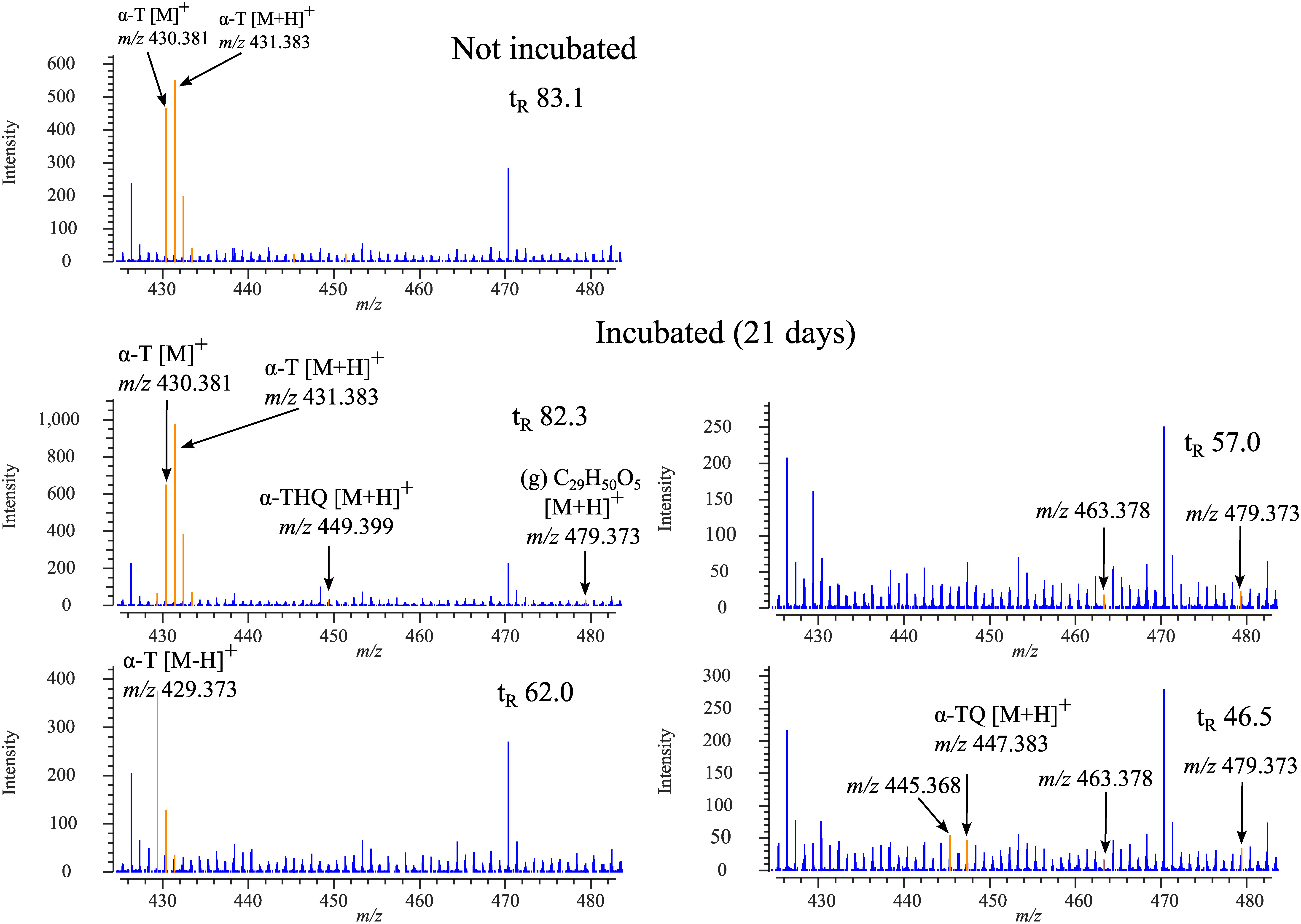
Fig. 9. Mass spectra at peak apex on the chromatograms shown in Fig. 8. The spectrum data files are available in J-STAGE Data. https://doi.org/10.50893/data.massspectrometry.21557259

Although split injection shows drastic improvement in column efficiency compared to splitless injection, extracted ion chromatograms for *m*/*z* 429.373 and 430.381 show an unresolved peak. The isomers, that are detected on the same *m*/*z* on the PTR MS must be resolved *via* chromatography to effectively apply the tandem MS technique to select a precursor ion. We have further evaluated the peak resolution using L-column3 C18 with a 2.1-mm i.d. and a 100-mm length. Figure 8 shows a comparison of α-T chromatograms for the freshly prepared sample (bottom) and 21 days incubated sample (top) obtained using L-column3 with split injection and 10 pmol of α-T sample applied to the SFE frit. No chromatographic peaks were detected at *m*/*z* 429.373, 447.383, or 445.368 in the freshly prepared sample. We observed several chromatographic peaks in extracted ion chromatograms at *m*/*z* 429.373, 447.383, and 445.363, which correspond to the α-T oxidative products in addition to 430.381 and 431.388 on 21 days of incubated samples as shown in Fig. 8 (top). By contrast, no chromatographic peaks except for *m*/*z* 430.381 and 431.388 were observed from freshly prepared α-T samples shown in Fig. 8 (bottom), although chromatograms for *m*/*z* 429.373, 447.383, and 445.363 were not plotted for better visibility. The peak parameters for all the detected peaks are listed in Table 2, and the mass spectra obtained at the peak apex are presented in Fig. 9.

**Table 2:** Table 2. Peak parameter for the peaks appeared on the chromatograms shown in Fig. 8.

*m*/*z*	*t*_R_(s)	Area	Height	Width(s)	NTP	*R*s	Asymmetry	k
429.373	57.0	8.95	3.37	3.46	1505		1.1	1.22
	62.0	22.52	8.82	2.44	3577	1.0	1.0	1.42
	82.5	2.08	0.66	2.29	7168	5.1	1.1	2.22
430.381	40.9	4.25	0.97	2.72	1255		2.4	0.59
	57.1	3.80	1.21	3.27	1691	3.2	1.0	1.23
	62.0	10.44	3.44	2.47	3484	1.0	1.1	1.42
	82.3	63.09	16.72	3.40	3244	4.1	1.5	2.21
431.388	62.2	2.85	0.96	2.69	2955		0.8	1.43
	82.4	103.27	29.19	3.17	3742	4.1	1.5	2.21
445.368	45.1	4.12	1.19	3.12	1159		1.1	0.76
	51.0	3.03	0.84	3.20	1407	1.1	1.9	0.99
447.383	46.5	5.14	1.05	3.09	1260		1.4	0.81
	56.9	4.99	1.43	3.44	1516	1.9	1.7	1.22
463.378	44.6	1.35	0.34	2.80	1408		1.4	0.74
	48.6	1.27	0.43	3.20	1279	0.86	1.5	0.90
479.373	44.5	1.78	0.44	2.69	1511		1.2	0.73
	49.5	1.46	0.36	2.69	1883	1.1	1.2	0.93
449.399	82.8	2.86	0.57	3.12	3895		1.2	2.23

An ion peak appeared at *m*/*z* 430.381, corresponding to the α-T-radical, was consistently observed alongside the protonated α-T ion at the same retention time of 82.3 s, without exception. Therefore, it might have been generated during the PTR ionization process.

Two prominent peaks were observed for *m*/*z* 429.373 at 57.0 and 62.0 s, besides 82.5 s. These two peaks were not shown on a freshly prepared sample (Fig. 8, bottom), thus they were considered as incubation products, although we do not have any information to assign them to (b), (c), or something else.

Two peaks observed for *m*/*z* 430.381 at 57.1 and 62.0 s may correspond to an isotope of *m*/*z* 429.373.

Two peaks with retention times of 46.5 and 56.9 s appeared on an *m*/*z* 447.383 chromatogram are supposed to be molecules (d) and (f). The log *P* value suggests that α-TQ (f) may exhibit a shorter retention time than molecule (d). Therefore, the 46.5-s peak at *m*/*z* 447.383 is labeled as α-TQ in Fig. 8 (top).

A peak observed at *m*/*z* 449.399 at 82.8 s listed in Table 2 bottom may correspond to α-tocopherylhydroquinone. One of the two peaks observed for *m*/*z* 479.373 may be ascribed to the molecule (g), and one of the peaks observed at *m*/*z* 463.378 may correspond to 5-formyl-γ-tocopherol+OH, according to Tang *et al.*^19)^

All the peaks appearing on the chromatogram at the same *m*/*z* are separated by a peak resolution (*R*s) of at least 0.86, making it possible to select precursor ions for tandem MS. We are working to reduce the instrumental dispersion at the PTR flow tube, which is currently approximately two seconds. We aim to minimize the dispersion that gives a minimum peak width of less than one second so that all the peaks listed here are fully resolved at *R*s>1.5.

The analysis of fat-soluble vitamins in the biological samples is not a trivial issue in liquid chromatography but it has been done well by using SFC.^22,23)^ The analysis of tocopherols in natural products such as wheat germ or vegetable oil by using SFE/SFC was also reported in past decades.^24)^ However, the analysis of α-T oxidative product has been studied since it was extremely complex and is a short lifetime. Since it is a non-enzymatic reaction, the enzyme kinetics study is not applicable to studying plant self-defense systems from ^1^O_2_. Although several issues need to be addressed such as peak dispersion in the ion source, the present method is great potential to study plant physiology.

## 4. CONCLUSION

We have evaluated the SFE/SFC–PTR MS to separate α-T and its oxidative products that play a crucial role in the antioxidant defense system of a plant cell during photosynthesis. The mechanism and reaction scheme of α-T antioxidant function have been recently investigated using multiple analytical instruments, including chromatographic separation and complex sample processing. The combination of SFE with SFC and PTR MS has achieved almost the complete peak resolution of isomers reported in the literature in less than 2 min of chromatography by simply applying the sample to a stainless steel frit. Structure determination still requires the use of tandem MS to identify molecules; isomers must be resolved before ionization to select precursor ions. The present study demonstrated that SFE/SFC split injection can effectively separate α-T oxidative products without requiring dedicated sample preparation techniques such as solid-phase extraction. This method achieved a fast chromatography separation rate with high detection sensitivity and exhibits excellent potential for analyzing complex sample matrices, including cells and tissues. It may help detect molecules from a single cell or small amounts of tissues.

## Data Availability

The spectrum data files of Fig. 9 are available in J-STAGE Data.

## Notes

Mass Spectrom (Tokyo) 2022; 11(1): A0108
